# Idiopathic Sudden Sensorineural Hearing Loss: Average Time Elapsed Before Presentation to the Otolaryngologist and Effectiveness of Oral and/or Intratympanic Steroids in Late Presentations

**DOI:** 10.7759/cureus.1945

**Published:** 2017-12-14

**Authors:** Anwuli Anyah, Devin Mistry, Erin Kevern, Kenneth Markiewicz

**Affiliations:** 1 Otolaryngology, Metro Health Hospital - University of Michigan

**Keywords:** sudden hearing loss, intratympanic steroids, time to presentation, sudden sensorineural hearing loss, sudden idiopathic sensorineural hearing loss, steroids and hearing loss

## Abstract

Objectives

To determine how long after symptom onset that the average patient with an idiopathic sudden sensorineural hearing loss (ISSNHL) presents to the otolaryngology clinic.

In late presentations, to determine the time to presentation cutoff after which intervention may not be effective.

To evaluate the effectiveness of oral steroids versus a combination of oral and intratympanic steroid therapy in late presentations of ISSNHL.

Methods and procedures

Sixty-four patients met inclusion criteria after chart review of 2,037 patients seen at Metro Health Hospital from 2006 to 2016 for sensorineural hearing loss. All sixty-four patients were used to calculate the average time to presentation, but only 40 were included to evaluate treatment efficacy because 24 were lost to follow-up or declined treatment. Audiograms were analyzed for baseline status and response to treatment. Therapy was either oral steroids or intratympanic (IT) steroids. Thirty-nine of the 40 treated patients received oral steroid therapy. Eighteen of these 39 patients received both oral and IT steroids. One patient received IT steroids only.

Results

For all 64 patients in the study, the average time to presentation was 55 days, ranging from one day to 240 days. Data for 32 of the 40 treated patients were analyzed. These patients were further divided into smaller groups: Group 1 (N = 11) - treatment within seven days of symptom onset, Group 2 (N = 17) - time to treatment greater than seven days but less than 90 days of symptom onset, and Group 3 (N = 4) - greater than 90 days of symptom onset. In Group 2, there was a significant improvement in pure tone average (P-value: 0.005). Forty-seven percent of patients in this group had objective treatment response utilizing Wilson’s criteria. Two patients had a complete recovery and six had a partial recovery. Hearing gains ranged from 10 dB (decibels) to 23 dB. Sixty-three percent of patients with objective improvement also had subjective improvement. In Group 3, none of the patients met Wilson’s criteria for recovery. There was no statistically significant difference in response between patients treated with oral steroids only versus a combination of oral and IT steroids.

Conclusion

Patients with ISSNHL present to an otolaryngologist on average 55 days after symptom onset. There is statistically and clinically significant response to treatment in late presenters. Improvement can be seen up to three months from symptom onset. Oral steroid therapy is effective. IT steroid therapy may have an added benefit.

## Introduction

Idiopathic sudden sensorineural hearing loss (ISSNHL) is defined as sensorineural hearing loss of 30 decibels (dB) or more over at least three contiguous audiometric frequencies with an onset of fewer than three days. This definition is often used in order to be consistent with the literature and the National Institute on Deafness and Other Communication Disorders criteria. In clinical practice, however, it is often reasonable and necessary to expand the definition to cases with less than 30 dB of hearing loss [1]. The incidence of ISSNHL is approximately five to 20 per 100,000 population [2]. The etiology and pathogenesis of ISSNHL remain unknown. Proposed theories include vascular compromise, labyrinthine membrane ruptures, a viral infection of the cochlea, etc. Saumil, et al. histologically examined 17 temporal bones with ISSNHL [3]. No histologic correlates were found in two ears that recovered hearing after treatment. In the remaining 15 ears, the predominant abnormalities included loss of hair cells and supporting cells of the organ of Corti, loss of the tectorial membrane, loss of stria vascularis, and loss of cochlear neurons.

From the few good studies available, it is generally accepted that early intervention in ISSNHL may increase recovery [1]. It is well known that patients who start treatment within seven days of symptom onset have a higher rate of recovery [4]. According to the current American Academy of Otolaryngology-Head and Neck Surgery (AAOHNS) clinical practice guidelines, oral steroids, intratympanic (IT) steroids, and hyperbaric oxygen therapy are the recommended treatment options for ISSNHL [1].

In the general practice of an otolaryngologist, a significant number of patients with ISSNHL present more than seven days after symptom onset. This is because a common initial symptom of ISSNHL is aural fullness or a plugged feeling in the affected ear [1]. Because this is a nonspecific symptom, primary care physicians and patients are unalarmed, often causing a delay in appropriate treatment initiation [1]. General practitioners often initiate antibiotics or a steroid nasal spray and only refer to a specialist after no improvement is seen. 

There are few studies dedicated to evaluating the effect of time to therapy initiation; most of these studies contain a good proportion of patients that present less than seven days from symptom onset, which is within the recommended time frame for treatment. Upon literature review of the last five years of research on sudden hearing loss in adults, no studies have been formally dedicated to evaluating the average time between symptom onset and presentation in ISSNHL. Few studies have been dedicated to examining the effect of time passed before treatment initiation. Even fewer studies have been dedicated to examining the response to treatment in late presenters. No study was found that simultaneously compared the effectiveness of oral steroids and intratympanic steroids in late presentations (greater than seven days from symptom onset). Interestingly, the studies that evaluated the effects of time elapsed before treatment had contrasting results. Marco, et al. showed that time elapsing between symptom onset, the start of therapy, and duration of treatment was not a factor in hearing recovery [5]. Bogaz, et al. studied the response of oral steroid treatment as it relates to the time elapsed prior to treatment initiation [4]. They demonstrated that response was better when treatment was initiated early (within seven days), but their study was limited because it only evaluated response to oral steroids. 

The objectives of this study are to 1) determine how long after symptom onset the average patient with ISSNHL presents to the otolaryngology clinic; 2) in late presentations, to determine if there is a time to presentation cutoff after which intervention may not be effective; and 3) to study the effectiveness of oral steroids versus a combination of oral and intratympanic steroid therapy in late presentations of ISSNHL. The ultimate goal is to educate clinicians on managing patients with ISSNHL who present late, as is often the case in the typical otolaryngology clinic.

## Materials and methods

There were a total of 64 patients involved in the study. This number was attained after a chart review of 2,037 patients seen at the Metro Health Hospital from the year 2006 to 2016 for sensorineural hearing loss. These 64 patients met the inclusion criteria of this study. Of these, 26 were male and 38 were female. The average age of presentation was 54 years. Thirty-five patients reported left-sided hearing loss and 28 reported right-sided hearing loss. 

Inclusion criteria for the study were an age of at least 18 years and the diagnosis of ISSNHL. The hearing loss had to be of unknown etiology, rapid onset - occurring over a 72-hour period, and affect at least three contiguous frequencies on audiometry. Exclusion criteria were the presence of middle and inner ear disease that could explain the hearing loss, such as trauma, infection, perilymphatic fistula, neoplasm or retro-cochlear lesion, congenital cochlear malformation, degenerative disease of the central nervous system, prior episodes of sudden hearing loss or fluctuating hearing loss (Meniere’s disease), focal neurological findings associated with the hearing loss, or ischemic brain lesions.

All patients included in the study had a type A tympanogram. Data collected from patients were age, sex, laterality of hearing loss, estimated date of hearing loss, date of presentation, baseline audiogram, pretreatment tympanogram, pretreatment speech discrimination scores (SDS), pretreatment audiogram, accompanying symptoms, date of treatment onset, type of treatment with doses, and duration of treatment. 

Audiograms were analyzed for baseline status and response to treatment. If low and medium tones were involved in the hearing loss, the means of 0.25, 0.5, 1, and 2 kHz frequencies were calculated. When medium and high frequencies were affected, the means of 1, 2, 3, 4, 6, and 8 KHz were calculated. When only high frequencies were affected, the means of 3, 4, 6, and 8 kHz were used [4]. The audiology baseline for each patient was calculated as the average of the pure tone averages (PTA) from a pre-ISSNHL audiogram of the frequencies affected. In patients without a baseline audiogram, the audiogram results of the non-affected ear were utilized as a baseline.

Treatment in the study population was either oral steroids or intratympanic steroids. Thirty-nine of the 40 patients received oral steroid therapy. Eighteen of these 39 patients also received intratympanic steroids. A prednisone taper was the most common oral steroid therapy. Patients were instructed to take 60 mg for nine days and tapered over the following five days for a total of 14 days. This regimen was utilized in 36 of the 39 patients who opted for oral steroid therapy. One of the 39 patients was treated with a Medrol® Dosepak™ (Pfizer, Inc., New York, NY), one with decadron 8 mg every eight hours for seven days, and one patient was treated with decadron in a tapering fashion unique from the other patients in the study. It was administered 6 mg on day one, 4.5 mg on days two to four, 3.75 mg on days five to seven, 3 mg on days eight and nine, 1.5 mg on days 10 and 11, and 0.75 mg on days 12 and 13. 

Of the 18 patients who were treated with both oral and IT steroid therapy, 15 were treated with weekly sessions of IT steroids. Under a microscope, topical phenol was used to anesthetize a portion of the tympanic membrane (TM) and 0.5 ml of dexamethasone (4 mg/ml) was injected through the anesthetized area. The patient was kept in recumbent position for 15 minutes after the procedure. The number of sessions varied per patient depending on symptom resolution or willingness to continue therapy. The average number of sessions was 2.6 with a range of one to five sessions. The remaining three patients had tympanostomy tubes placed and administered 0.1% decadron ophthalmic suspension, six drops to the affected ear twice daily for two weeks. Regardless of the method of delivery, IT steroids were started on average nine days from beginning oral steroid therapy with a range of zero to 30 days. The only patient that was treated with solely IT steroids also had a tympanostomy tube placement and decadron drops in the same dosage described above.

Many studies devoted to ISSNHL do not have clearly defined endpoints. This was avoided in this study as evidenced by subsequently described criteria. Response to therapy was deemed significant if it met the internationally accepted Wilson’s criteria which are as follows: (1) Complete recovery is post-treatment PTA within 10 dB of baseline audiogram; (2) partial recovery if post-treatment PTA is within 50% of initial hearing level or > 10 dB improvement in hearing; or (3) no recovery is a < 10 dB improvement [1, 5-8].

The data collected were statistically analyzed using "paired t-test", also called a "dependent t-test”. The Statistical Package for Social Sciences (SPSS), Version 20 (IBM SPSS Statistics, Armonk, NY) was utilized for the analysis.

## Results

All 64 patients were used to calculate the average time to presentation, but only 40 were included to evaluate the treatment effects because 24 were lost to follow-up or declined treatment. The average time to presentation was 55 days with the earliest presentation being one day and latest presentation being 240 days. 

For the treatment group, there were 40 patients in total (see Table 1 for statistics). The average age was 54 years, with the youngest age being 25 and oldest being 81 years. This group had 24 female and 16 male patients. Twenty-five complained of left-sided hearing loss and 15 complained of right-sided loss. The average time to presentation was 36 days with a range of one day to 189 days. The average time to treatment was essentially the same as time to presentation (Table 1). Thirty-nine patients were treated with oral steroids, and 18 were treated with a combination of oral and intratympanic steroids. Only one patient was treated with intratympanic steroids only. No patient was treated with hyperbaric oxygen.

**Table 1:** Descriptive Statistics Std: standard; dB: decibel

Descriptive Statistics					
	N	Minimum	Maximum	Mean	Std. Deviation
Age	40	25	81	53.97	16.633
Days elapsed before presentation	40	1	189	36.03	47.789
Baseline audiogram (dB)	39	0	94	22.08	20.241
Pretreatment audiogram (dB)	35	20	118	65.63	24.324
Days elapsed before treatment	40	1	189	36.05	47.793
Duration of treatment	40	7	42	17.42	6.808
Post-treatment audiogram	34	9	108	56.29	26.84

Data for 32 of the 40 treated patients were statistically analyzed because six had no response on pre and/or post-treatment audiograms; hence, a value could not be assigned to the audiogram results. One patient’s audiogram showed a conductive loss (implying an incorrect diagnosis), and one patient had no post-treatment audiogram. The treated patients were further divided into groups for statistical analysis: Group 1 (N = 11) - treatment within seven days of symptom onset, Group 2 (N = 17) - time to treatment greater than seven days but less than 90 days of symptom onset; and Group 3 (N = 4) - greater than 90 days from  symptom onset. The actual range for this group is 90 to 189 days. Table 2 lists the statistical data for each treatment group.

**Table 2:** Paired Samples Statistics for Groups 1, 2, and 3 dB: decibel; Std: standard

Paired Samples Statistics					
		Mean (dB)	N	Std. Deviation	Std. Error Mean
Group 1	Pre-treatment Audiogram	66.55	11	28.668	8.644
	Post-treatment Audiogram	50.09	11	29.002	8.744
Group 2	Pre-treatment Audiogram	64.18	17	25.108	6.090
	Post-treatment Audiogram	57.06	17	28.374	6.882
Group 3	Pre-treatment Audiogram	66.75	4	16.235	8.118
	Post-treatment Audiogram	69.75	4	19.670	9.835

Group 1’s outcome was as expected. The mean PTA improvement was 16.46 dB with a standard deviation of 13.8 dB. This was a significant improvement (p-value: 0.003). In Group 2, there was a significant improvement in PTA (p-value: 0.005). Of the patients in Group 2, 47% (N = 8) had an objective treatment response utilizing Wilson’s criteria. Of the patients that responded to treatment, two patients had a complete recovery and six had a partial recovery. The average PTA gain was 7.12 dB with a standard deviation (STD) of 9.0 dB for the group as a whole. Hearing gains in this group ranged from 10 dB to 23 dB (Tables 3-4). It is important to note that 63% of patients that had measurable hearing improvement also had subjective improvement. In Group 3, none of the patients met Wilson’s criteria for recovery. 

**Table 3:** Paired Differences in Pre- and Post-treatment Audiograms for Groups 1, 2, and 3 dB: decibel; Std: standard; t: t-test statistic; df: degrees of freedom

Paired Differences in Pre and Posttreatment Audiograms								
	Mean (dB)	Std. Deviation	Std. Error Mean	95% Confidence Lower	95% Confidence Upper	t	df	p-value
Group 1	16.455	13.823	4.168	7.168	25.741	3.948	10	0.003
Group 2	7.118	8.957	2.172	2.512	11.723	3.276	16	0.005
Group 3	-3.000	4.163	2.082	-9.625	3.625	-1.441	3	0.245

**Table 4:** Detailed Subject Response to Treatment ID: identification; PTA: Pure tone average; dB: decibel; N: no; Y: yes

Subject ID #	Time Elapsed Before Presentation and Treatment (Days)	Baseline Audiogram (PTA in dB)	Pre-treatment Audiogram Results (PTA in dB)	Post-treatment Audiogram Results (PTA in dB)	PTA Improvement	Patient's Subjective Perception of Hearing Recovery	Treatment modality		Duration of Treatment (Days)	Wilson's Category
							Oral Steroids	Intratympanic Steroids		
2	52	29	45	42	3	Not Improved	Y	N	7	
4	12	15	105	95	10	Improved	Y	Y	14	Partial Recovery
8	15	66	103	80	23	Improved	Y	N	14	Partial Recovery
10	47	13	63	63	0	Not Improved	Y	Y	14	
15	19	10	98	94	4	Not Improved	Y	Y	21	
17	60	8	38	16	22	Improved	Y	Y	42	Complete Recovery
20	30	36	71	76	-4	Improved	Y	Y	21	
24	23	32	103	108	-5	Not Improved	Y	Y	21	
25	14	24	54	54	0	Improved	Y	Y	28	
28	44	12	52	41	11	Not Improved	N	Y	14	Partial Recovery
29	83	37	70	69	1	Not Improved	Y	Y	14	
30	74	25	58	48	10	Not Improved	Y	Y	14	Partial Recovery
33	9	7	20	9	11	Improved	Y	N	14	Complete Recovery
36	30	12	49	47	2	Not Improved	Y	N	14	
37	21	12	42	21	21	Improved	Y	Y	25	Partial Recovery
39	30	0	52	39	13	Not Improved	Y	Y	21	Partial Recovery
40	30	12	68	68	0	Not Improved	Y	N	14	

On analyzing the modes of treatment utilized in the late presenters (Groups 2 and 3), the average PTA before treatment of those that received oral steroids (N = 21) was 69.48 dB and after treatment was 61.67 dB. This comes out to be 7.8 dB improvement with a STD of 13.186 dB and of statistical significance (p-value: 0.013). In the group that received both oral and intratympanic steroids (N = 13), the average PTA before treatment was 70.62 dB and after treatment was 64.77 dB. This comes out to be 5.846 dB improvement with a STD of 9.822 dB. The difference found in this group was not statistically significant (p-value: 0.053) (Table 5).

**Table 5:** Paired Differences in Pre- and Post-treatment Audiograms for Treatment Modalities dB: decibel; Std: standard; t: t-test statistic; df: degrees of freedom

Paired Differences in Pre- and Post-treatment Audiograms									
	N	Mean (dB)	Std. Deviation	Std. Error Mean	95% Confidence Lower	95% Confidence Upper	t	df	p-value
Oral Steroids only	21	7.81	13.166	2.877	1.807	13.812	2.714	20	0.013
Oral and Intratympanic Steroids	13	5.846	9.822	2.724	-0.089	11.782	2.146	12	0.053

There were a few adverse effects of treatment. Five of the 14 patients that received IT steroids developed TM perforations, but only one patient required a tympanoplasty. One patient that received both oral and IT steroids developed an exacerbation of a preexisting anxiety disorder.  

## Discussion

Although idiopathic sudden sensorineural hearing loss (ISSNHL) is defined as sensorineural hearing loss of 30 dB or more over at least three contiguous audiometric frequencies and an onset of fewer than three days, it is acceptable and often necessary to expand the definition to cases of sudden hearing loss that is less than 30 dB [6]*.* Hearing loss that is appreciable to the patient and measurable can and should be included in the definition so as to offer treatment accordingly. 

The treatment response in Group 1 is as expected and has been shown in papers that studied treatment response in ISSNHL. One of the oldest studies that investigated the response to oral steroids by Wilson, et al. in 1980 was performed in patients that presented within 10 days of symptom onset [8]. They found a statistically significant response in the oral steroids vs placebo group. Another study by Bogaz, et al. showed that patients who started treatment before seven days had higher rates of recovery compared to later presenters [4]. In Group 2, which is this study’s main interest, there was a significant improvement in PTA (p-value: 0.005). Forty-seven percent (N = 8) had objective treatment response utilizing Wilson’s criteria. Of the patients that responded to treatment, two patients had a complete recovery and six had a partial recovery. Average PTA gains were 7.12 dB with a STD of 9.0 dB for the group as a whole. Hearing gains in this group ranged from 10 to 23 dB. The response seen in this group is impressive, given that these patients presented very late (up to 90 days after symptom onset). Again, it is important to note that 63% of patients that had measurable hearing improvement also had subjective improvement. This demonstrates that this statistical significance is also clinically significant.

No hearing improvement was observed in Group 3. This is not surprising given that this group contains patients that presented extremely late. The exact range of presentation timing for this group ranged from 90 to 189 days. These findings suggest that although it is reasonable to attempt treatment in all patients (given the low risk of the treatment and the potential of hearing improvement), treatment may not be effective if the initial insult is greater than 90 days. 

In the recent literature, there has not been a study like this that evaluated the effect of initial treatment on late presenters. The closest study to this was one study that evaluated the effect of re-treatment of end-stage sudden deafness [9]. Given that it was a re-treatment study, the time to therapy was between two to six months. There was a 46.43% response and average 7.12 dB gains, and this improvement was considered a success. The treatment regimen in that study was a combination of sodium bicarbonate, dexamethasone, and batroxobin 5BU administered intravenously. 

The findings in this paper suggest that there is a benefit in treating ISSNHL patients that present late, especially if the time of onset is within 90 days. There was a statistically significant improvement in hearing in this group of patients, and a majority of patients who responded to treatment also had subjective improvement in hearing. The hearing gain seen could be the difference between different categories of hearing loss and may bring the patient to serviceable hearing levels. 

On analyzing the group of patients who also received intratympanic steroids, it is interesting to see that although PTA improvement was seen and almost comparable in magnitude to that of the oral steroid only group, it was not statistically significant. This is likely multifactorial. One reason may be the small number of 13 patients in this group. Another reason could be that patients with a worse prognosis constituted this group. The majority of patients in this group were started on IT steroids after they had failed a trial of oral steroids. This is an area of limitation in this study. Being a retrospective study, there were limits in the study design. A study that compares oral vs. IT steroid therapy as initial treatment modalities is necessary to be able to effectively compare these modes of treatment. 

During the data collection stage of this project, an attempt was made to document associated symptoms, such as aural fullness, tinnitus, or vertigo, so as to study the resolution of these symptoms with therapy. This portion of the study could not be completed because data extracted from the subjective portion of patients' charts were often incomplete. These associated symptoms were not the chief complaints and hence not documented at every visit. This is another area of weakness in this retrospective study. In a prospective study, there would be an opportunity to make a concerted effort to keep track of symptoms in the form of a questionnaire that patients can complete at every visit.

As with any treatment, the risk of oral steroid therapy should be discussed with patients. Side effects of oral corticosteroids include insomnia, dizziness, weight gain, gastritis, mood changes, photosensitivity, and hyperglycemia. Severe but rare side effects include pancreatitis, bleeding, hypertension, cataracts, myopathy, opportunistic infections, osteoporosis, and osteonecrosis [1]. Treatment of ISSNHL with steroids is a relatively short course and most often well tolerated. In this study, the only adverse effect of oral steroid therapy was an exacerbation of a preexisting anxiety disorder in one patient. 

Select patients, like insulin-dependent or poorly controlled diabetics or patients with labile hypertension, tuberculosis, or peptic ulcer disease may not be able to tolerate oral steroid treatment [1]. IT steroid therapy is a good option for these patients.  Alexander, et al. [10] demonstrated that even in long-term steroid treatment (up to 22 weeks) for autoimmune ear disease, the most frequent adverse effect was hyperglycemia and weight gain. Severe events like osteonecrosis and fractures are more commonly seen in patients with preexisting bone and joint problems [11].

IT steroids have the advantage of having no systemic adverse effects. It is a good option for patients who cannot tolerate oral steroids. The most commonly cited adverse effect of IT steroids is TM perforations. Five of the 14 patients that received IT steroids in this study developed TM perforations. All patients recovered but one patient required a tympanoplasty. This experience is similar to that in the study by Slattery, et al. [12] where a 20% rate of TM perforations was reported.  All patients in that study were treated with patches and the perforation resolved within two weeks.

## Conclusions

Patients with ISSNHL often present late. The average time to presentation seen in this study was about 55 days. The authors advocate for treatment of patients with ISSNHL who present late, given that treatment is relatively safe. As evidenced in this study, there is a statistically and clinically significant response to treatment in late presenters. Hearing improvement was observed when treatment was initiated in patients that presented up to three months from symptom onset. Oral steroid therapy is effective. IT steroid therapy may have an added benefit.
